# Physical Activity Levels of 1053 Omani 4th Grade Children: The Importance of Gender and Sport Team Participation in Achieving 60 Minutes of Daily Moderate-to-Vigorous Physical Activity

**DOI:** 10.3390/ijerph18168504

**Published:** 2021-08-12

**Authors:** Marc Lochbaum, Jonathan Kenyon, Youngdeok Kim

**Affiliations:** 1 Department of Kinesiology and Sport Management, Texas Tech University, Lubbock, TX 79409, USA; 2 Education Academy, Vytautas Magnus University, 44248 Kaunas, Lithuania; 3 Department of Kinesiology and Health Sciences, Virginia Commonwealth University, Richmond, VA 23284, USA; kenyonjd@vcu.edu (J.K.); kimy13@vcu.edu (Y.K.)

**Keywords:** health behavior, elementary school children, sports participation, sedentary behavior

## Abstract

Sufficient daily physical activity is associated with many positive mental, physical, and societal benefits in children. Unfortunately, most children worldwide do not achieve recommended levels of daily physical activity (PA), and a majority of evidence is from Western countries and based on subjective measures. This study examined the prevalence and correlates of objectively measured PA levels among Omani children in 2017 (pre-pandemic). A two-stage cluster sampling was used to recruit the 4th grade children across five regions of Oman. A final analytic sample included 1053 children (504 boys, 549 girls) with a mean age of 9.21 years old. PA was objectively measured using a wrist-worn Polar Active Watch during three consecutive school days. Screen-based sedentary behaviors and other PA-related behaviors were subjectively measured. On average, boys were less sedentary and more active, with a greater likelihood of meeting current recommendations when compared with girls. The self-reported time spent in screen-based sedentary behaviors was relatively low for both boys and girls and was not associated with PA; however, sports team participation was associated with a greater likelihood of meeting the current recommendation. The present study provides empirical data on objectively measured PA in Omani children. The gender disparities concerning daily PA, including sports team participation, should receive further attention.

## 1. Introduction

Researchers have summarized decades of information corroborating that engagement in recommended amounts of physical activity unequivocally decreases susceptibility to many chronic diseases such as obesity, type II diabetes, and heart diseases [1,2]. To gain the protective benefits of physical activity, it is important to start physical activity at a young age. Research demonstrates that physical activity pursuits during childhood and adolescence set up a more positive trajectory for leisure time physical activities in adulthood [3,4]. Thus, getting children engaged in a sufficient level of physical activity at a young age is important.

The World Health Organization recommends that children engage in at least 60 min of moderate- and vigorous-intensity physical activity (MVPA) per day [1], with at least 30 min of MVPA occurring during after-school hours [5]. Unfortunately, a majority of children worldwide do not engage in a recommended amount of physical activity, with a decreasing trend of meeting the recommendation over time [6]. In addition, increasing trends of sedentary behaviors such as screen time in pediatric populations have become a growing concern due to the harmful effects of sedentary behaviors on obesity and cardiometabolic health [7]. Such a physically inactive lifestyle in childhood has become a critical health concern to the Gulf Cooperation Council (GCC) countries, including Oman [8], showing a rapidly increasing prevalence of obesity across the lifespan [9].

To date, there have been few studies examining the prevalence of physical activity and sedentary behaviors in Omani youth [10,11,12]. Most previous studies examining associations between lifestyle behaviors, physical activity, and sedentary behaviors in the youth of Oman and other GCC countries have used subjective measures such as self-report questionnaires that are less valid than objective measures such as accelerometry to assess accurate levels of physical activity [13]. Further, many of the previous studies have included a broad range of ages in their samples or only included adolescents without consideration for young and middle-aged children. Given that many health benefits such as increased bone mineral density occur just prior to and during the early stages of puberty [2] and healthy habits developed during middle childhood (6 to 11 years) positively influence sustainable changes years later [14], it becomes more important to assess the activity levels of middle-aged children. This highlights a need for additional studies using objective instruments to measure physical activity during middle childhood in Omani children.

Thus, the purpose of this study was to objectively examine the levels and patterns of physical activity in Omani children during school days using an accelerometer. We additionally examined the correlates of the objectively measured physical activity with self-reported measures of exercise and sedentary behaviors to better understand the potential factors influencing activity levels in Omani children. The results of this study will provide valuable baseline data to better inform and position policymakers for the development of intervention strategies against rising obesity in Omani children.

## 2. Materials and Methods

### 2.1. Survey Sample

A two-stage cluster sampling design was used to produce a sample of 4th-grade children attending public elementary schools in five urban city districts in Oman (Muscat, Salalah, Seeb, Sohar, and Sur). From each city district, five mixed-gender primary elementary schools were randomly selected. In the second sampling stage, two 4th-grade classes were randomly selected from each school, and all students in the randomly selected classes were eligible to participate in the study. The schools and families of eligible students randomly chosen at each stage were provided with an informed consent form, with the support of the Oman Ministry of Education, and all agreed to participate in the study. All schools offered physical education class every school day for a duration of 30 to 40 min by following the nationwide standard curriculum. The data were collected from October to December 2017. The participants were 1206 children, who participated across twenty-five schools, of which 146 were excluded due to noncompliance with the physical activity monitoring protocol (described later). Thus, the final analytic sample consisted of 1053 children (504 boys; 549 girls) with a mean age of 9.21 years old (95% CI = 9.19–9.24 years old). Informed school and parental consent were obtained prior to data collection along with the child’s verbal assent. The study protocols were reviewed and approved by the Texas Tech University Institutional Review Board (IRB2016-1104) and the Oman Ministry of Education.

### 2.2. Objectively Measured Physical Activity

Physical activity was objectively measured using a Polar Active (Polar Electro Inc, Kempele, Finland). The Polar Active is a lightweight, watch-style uniaxial accelerometer designed for children. The watch has a digital screen on its face displaying visual feedback on the user’s activity levels, which may positively motivate a child to be more physically active than usual. Thus, prior to distribution, we blocked the digital screen with a dark sticker in order to avoid potential motivational bias. Watch distribution occurred in a group setting (e.g., all students in the same room such as the library) at each school with assistance from school officials speaking the Omani language. On average, 50 devices were distributed to the students from each of the 25 schools. The children were asked to wear the device on their non-dominant wrist during waking hours for three consecutive school days.

The Polar GOFIT website (https://polargofit.com; accessed on 28 September 2017) along with the Polar Websync Software (version 2.8.3., Polar Electro, Kempele, Finland were used to initialize the device, and to download and transfer the data from the device for further analysis. The child’s sex, date of birth, height (cm), and weight (kg) were entered into the software, and the child’s activity levels were estimated in the form of metabolic equivalent (METs) at 30 s epoch length using the manufacturer’s proprietary algorithm. For this study, the accelerometer non-wear time was defined as a period with 30 consecutive minutes of no movement with an allowance of up to 1 to 2 incidental movements at a 30 s epoch. The child who provided the accelerometer data for at least 1 valid school day with 10+ h of wear time was considered valid and included in the analysis. The time spent in sedentary (<2 METs), light (2 to 3.99 METs), and MVPA (≥4 METs) [15] were estimated during all waking hours, school hours (8:00 am to 2:45 pm), and non-school hours (i.e., outside of school hours during accelerometer wear time). A previous study reported that the Polar Active watch showed moderate convergent validity for the estimation of physical activity levels in children when compared to other research-grade accelerometers [16].

### 2.3. Self-Reported Physical Activity and Screen-Time Behaviors

Children were asked to complete a questionnaire on their physical activity pursuits and screen-time behavior. The questions were derived from the Youth Risk Behavior Surveillance questionnaire conducted by the Center for Disease Control and Prevention of the United States [17]. Specifically, the children were asked to report the number of days they were physically active for a total of at least 60 min per day during the past seven days and the number of out-of-school sports teams (i.e., in a sport club) they participated on during the past year. The children were categorized into two groups for each variable (‘meeting recommendation’ vs. ‘not meeting recommendation’; and ‘no sports team’ vs. ‘1 or more sport team’). To assess sedentary behaviors, the children were asked to report the time spent watching television and using a computer (including playing on a computer or video games) on an average school day. The children were categorized into two groups for watching TV (‘<2 h/day’ vs. ‘2+ h/day’) and using a computer (‘<2 h/day’ vs. ‘2+ h/day’) in agreement with the recommendation by the American Academy of Pediatrics [18].

### 2.4. Weight Status

The child’s height (cm) and weight (kg) were measured using a standard scale by a trained school staff at each school (e.g., physical education teacher, on-site nurse) before the distribution of the accelerometer. The body mass index (BMI) was calculated and compared to the World Health Organization age- and sex-specific BMI standard deviation scores [19] to classify the children into different weight statuses (underweight, normal, overweight, and obese).

### 2.5. Data Analysis

Descriptive statistics of study variables were calculated and presented using mean (standard deviation) for a continuous variable and frequency (%) for a categorical variable. A three-level multilevel linear or generalized linear model was constructed to test gender differences in study variables after accounting for the clustered sampling design where children were nested within schools and schools were nested within regions [20]. For the comparison of continuous variables, we additionally calculated the standard Cohen’s d as a pseudo effect-size indicating the magnitude of differences. Cohen’s d was interpreted as small (<0.02), medium (<0.50), and large (≥0.80) [21]. When comparing objectively measured activity levels, total wear time was adjusted in the model. Lastly, the likelihood of meeting accelerometer-based MVPA recommendation was examined in relation to the child’s weight status (non-obese vs. obese), physical activity pursuits including self-reported meeting recommendation and sports team participation, and sedentary behaviors including watching television and using a computer. The multilevel generalized linear model based on a binary distribution with logit link function was used and the estimates were presented using odds ratios (OR) and 95% confidence intervals (CI). SAS v9.4 (SAS Institute, Carry, NC, USA) was used for all data analyses, and statistical significance level was set at *p* ≤ 0.05.

## 3. Results

Table 1 presents the descriptive statistics of the children who participated in the study. There were 1053 children with nearly an equal number of boys and girls. The boys and girls were similar concerning all anthropometric measures except for height, with boys being slightly taller than girls. Overall, 15.19% and 15.67% of the children were overweight and obese, respectively. The frequency distribution of weight status was not statistically different by gender; however, boys showed a higher prevalence of obesity (18.06%) when compared with girls (13.48%).

The objectively measured physical activity levels are presented in Table 2. On average, children wore the accelerometer 13.75 h/day during the monitoring school days. More than 70% of waking hours were spent in sedentary (584.09 min/day) followed by light-intensity physical activity (181.72 min/day; 22.02% of total wear time). Total MVPA minutes (59.39 min/day) were accumulated nearly equally across school (MVPA = 27.95 min/day; 47.06%) and non-school hours (31.43 min/day; 52.94%). The results stratified by gender showed that boys spent significantly less time in sedentary but also greater time in light-intensity physical activity and MVPA when compared with girls during both school and non-school hours (*p*-Value < 0.001).

Table 3 shows the prevalence of meeting the current recommendations, ≥60 min of MVPA for all-day and ≥30 min of MVPA per day during school hours, based on the objectively measured physical activity data. Overall, 41.41% and 37.51% of children met the current recommendations during the entire day and school hours, respectively. When stratified by gender, more than half of the boys met the recommendations, which were significantly greater when compared with girls showing the lower prevalence of meeting the recommendations (≈25%).

As shown in Table 4, most children reported watching TV (79.30%) and using a computer (86.98%) for one hour or less during school days, with girls being more likely to report less time spent TV watching as well as using a computer than boys (*p*-Values < 0.001). Additionally, girls were less likely to participate in a sports team outside of school than boys (55.43% vs. 74.85% for girls and boys, respectively). For both boys and girls, the self-reporting of MVPA for at least one hour a day were in line with the accelerometer data, with boys more likely than girls to self-report one hour of activity a day.

The results of multilevel logistic regression predicting the likelihood of meeting objectively measured PA recommendation is present in Table 5. Overall, children who self-reported meeting current MVPA recommendation (OR = 1.37; 95% CI = 1.01, 1.86) and participated in 1 or more sports teams in the last year (OR = 1.40; 95% CI = 1.03, 2.90) showed a greater likelihood of meeting objectively measured physical activity recommendation. When stratified by gender, however, only sports team participation remained a significant predictor (OR = 1.72; 95% CI = 1.08, 2.73) in boys, and no significant associations were observed in girls.

## 4. Discussion

This study objectively examined the levels of physical activity during school days among a large sample of Omani children. We found that, on average, nearly 60% of children did not engage in sufficient MVPA to meet the current recommendation (≥60 MVPA min/day). Further analyses revealed that only 37.51% of children engaged in ≥30 MVPA minutes per day during school hours. The present study also demonstrated that Omani children spent a large portion of waking hours in sedentary activities; yet, interestingly, self-reported time spent in media uses (watching television and using a computer) were relatively low. Notably, there were significant gender disparities in physical activity levels in that girls were more physically inactive with greater time spent in sedentary than their counterpart boys.

To date, only a few countries in the GCC (i.e., UAE and Qatar) have publicly joined in the globally organized research literature for the fight against physical inactivity known as “The Global Matrix” [22]. In their reports, children scored very low on physical activity. Based on self-reports, less than 20% of children in both countries meet the recommended 60 min a day of MVPA. Another study based on self-report data from Saudi Arabia indicates that 40% of Saudi children engage in a sufficient amount of physical activity [23]. Although direct comparison is limited due to the different measures of physical activity used and age variations, our findings indicate that Omani children have higher levels of MVPA when compared with other GCC countries, even when compared against the objectively measured data from the US, in which 42.5% of children aged 6–11 years old met the current recommendation [24]. However, it should be noted that the prevalence of children engaging in a sufficient level of MVPA during school hours was relatively low (37.51%). A school is the most critical environmental setting influencing physical activity levels among school-aged children [25]. Over 90% of school-aged Omani children attend schools [26] where they spend nearly half of their waking time in a day. Taken together, given the crucial role of school settings to promote physical activity in children, more efforts to develop and implement multi-component, evidence-based interventions in the school setting should be encouraged in Oman [8].

Concerning self-reported sedentary behaviors, the collected data from the present study and the UAE are relatively close in that children self-report mostly limited TV and computer usage, whereas in Qatar, about 27% of children engaged in less than two hours of TV and computer time a day, which is still lower compared with their counterparts in Western countries [27,28]. Screen-based sedentary behaviors, including watching television and working on a computer, are the dominant behaviors accounting for a majority of sedentary time in children [29]. In this regard, the present results showing the relatively low prevalence of engaging in screen-based sedentary behaviors in Omani children are encouraging. However, given that the objectively measured sedentary time still accounts for a relatively high portion of waking hours, particularly outside of school hours, further research is warranted to better understand the nature of sedentary behaviors, specifically for Omani children.

In our sample, 13.29% of boys and 16.94% of girls were overweight, while 18.06% of boys and 13.48% of girls were obese. This is in contrast to a recent review reporting the mean rates of overweight and obesity from three studies in Saudi, UAE, and Kuwaiti 6- to-10-year-olds, which found that 14.2% of boys and 25.0% of girls were overweight or obese [29], as well as a previous study of Omani children, reporting that 17.4% of the children were classified as overweight or obese [12]. The previous studies in GCC countries have reported a lack of physical activity and a growing prevalence of highly sedentary behaviors as critical lifestyle behaviors increasing the risk of childhood obesity [30,31]; however, limited research investigating this relationship has included Omani children. In our study, weight status did not predict meeting recommended amounts of MVPA, which was unexpected given the large body of evidence demonstrating the associations between obesity and physical activity [32]. However, our findings are still consistent with a recent study of children from five Omani provinces [33] and Saudi Arabia [34]. The underlying factors that might influence the lack of association between obesity and physical activity in the present study include the failure to account for dietary behaviors as well as the cross-sectional nature of the study design [32,35]. Additional research is needed to further elucidate the relationship between obesity and physical activity in this population.

We found that there were significant gender disparities in physical activity levels in Omani children. Compared with boys, girls had significantly higher objectively measured daily sedentary minutes and lower light-intensity physical activity and MVPA minutes, for both school and non-school hours. As a result, the likelihood of meeting the MVPA recommendations was significantly lower for girls compared with boys. Such gender differences in activity levels may be due to gender-specific barriers and cultural norms such as conservative social norms, cultural expectations, and lack of teacher support for girls [36]. Previous studies have found that women in Arab countries perceive fewer opportunities to engage in physical activity (i.e., limited access to sports and exercise facilities) and disapproval from family or the community for exercising [37,38,39,40]. Additional research is necessary to understand how complex geographical barriers and social-cultural factors may contribute to an environment that is less conducive to physical activity for Omani girls. Nevertheless, the gender disparities observed in our study are also in agreement with several other studies worldwide, which generally found that boys performed more physical activity and had less sedentary time than girls [41]. However, when self-reported measures were examined, girls in our sample spent less time watching TV and using computers but were less likely to participate in sports or meet MVPA recommendations than boys. This may suggest that girls in our sample underreported their sedentary behaviors, although we cannot rule out the possibility that girls may have spent more time in other sedentary behaviors besides TV viewing and computer use than boys in our sample, which could explain the contradictory findings between our objective and subjective sedentary behavior measures. We recommend that Oman elementary schools incorporate intentional physical activity within the school day, in addition to physical education classes and unstructured play such as recess, to insure a minimum level of intense movement. For example, five minutes of intentional play (e.g., jumping jacks, hopping, and skipping) every hour for 6 h is equal to 30 min of additional activity.

### Implications and Limitations

Our analysis revealed that participating in sports teams predicted meeting recommended amounts of MVPA for boys only. This highlights the importance of participating in team sports for boys and the need for Oman elementary schools to consider offering small-sided sports games such as football, which offer great opportunities for MVPA if structured properly with encouraging adult leaders. Our findings suggest that girls may require more encouragement to meet MVPA guidelines. It may be more effective if this encouragement comes from a physically competent female adult teacher or coach who can act as a leader and as a role model (i.e., who engages herself in daily physical activity as well as participating in activities with the children). Elementary schools must provide equal opportunities for girls for physical activity and do so intentionally with competent adults. In the meantime, the present study also demonstrated that self-reported TV viewing and computer use did not predict meeting recommended amounts of MVPA, which is consistent with the previous studies in the literature [42,43]. This indicates that reducing screen-based sedentary behaviors may not be an effective strategy in Omani children who also have relatively low levels of watching television and using a computer. Physical activity is a complex behavior influenced by multiple interpersonal factors, and thus, future studies should focus on multi-level factors influencing physical activity, particularly for girls, that can be targeted for the development of future interventions.

Though our research was unique in Oman and other GCC countries, it is not without limitations. First, although our sampling scheme was based on a simple random sampling within each cluster (i.e., city district and school), minimizing potential sampling bias, we could not calculate the sampling weights due to the unknown target population size. Further, the data collections were conducted during school days only, and thus, caution is needed when generalizing the present study to population-level parameters and beyond typical school days. Second, the survey was cross-sectional and did not measure any potential factors linking PA levels and screen-based sedentary behaviors in Omani children. Future study is recommended to explore multilevel socioecological determinants based on a longitudinal observation. Third, the previous studies demonstrated acceptable reliability and validity of the Polar Active Watch in this age group; however, we cannot completely rule out potential bias in the estimated PA levels due to the measurement errors, particularly sedentary time. Future studies should incorporate a posture-classification device (e.g., activPAL) to better assess the time spent in sedentary behaviors. Lastly, screen-based sedentary behaviors were subjectively measured with a lack of variety in screen-media devices. A more robust approach (e.g., time diary and mobile application) that measures a wide range of screen-media devices (e.g., mobile devices) should be considered in future research.

## 5. Conclusions

In conclusion, we found that the majority of Omani children are not meeting physical activity recommendations for both in-school and out-of-school hours during school days. Girls are less active than boys and spend more time in sedentary pursuits; yet, both boys and girls self-reported to engage in less time in screen-based sedentary behaviors. Sports team participation was found to positively predict the likelihood of meeting physical activity recommendations in boys. Future studies examining the predictors of physical activity in girls and reducing the gender disparities in activity levels are warranted.

## Figures and Tables

**Table 1:** Descriptive statistics of children’s characteristics.

	Total	Boys	Girls	*p*-Value ^a^
*n*(%)	1053 (100%)	504 (47.86%)	549 (52.14%)	-
Age (years)	9.21 (0.43)	9.22 (0.44)	9.21 (0.42)	0.993
Height (cm)	130.67 (6.64)	131.10 (6.79)	130.28 (6.49)	0.027
Weight (kg)	30.14 (8.70)	30.23 (8.83)	30.06 (8.60)	0.597
BMI (kg/m^2^)	17.51 (4.18)	17.43 (4.07)	17.60 (4.29)	0.732
Weight status (*n*, %) ^b^				0.282
Underweight	97 (9.21%)	48 (9.52%)	49 (8.93%)	
Normal	631 (59.92%)	298 (59.13%)	333 (60.66%)	
Overweight	160 (15.19%)	67 (13.29%)	93 (16.94%)	
Obese	165 (15.67%)	91 (18.06%)	74 (13.48%)	

Values are mean (standard deviation) and *n* (%) for a continuous and categorical variable, respectively. BMI = body mass index. ^a^ *p*-Value is for the gender difference estimated from a three-level multilevel linear or generalized linear model accounting for the clustered sampling design (i.e., children nested within schools and school nested within regions). ^b^ weight status was determined based on the World Health Organization’s age- and sex-specific BMI.

**Table 2:** The Objectively Measured Physical Activity Levels in Omani Children During School Days.

	Total (*n* = 1053)	Boys (*n* = 504)	Girls (*n* = 549)	*p*-Value ^a^	Cohen’s *d* ^b^
**All-day (waking hours)**					
Wear time (h/day)	13.75 (1.17)	13.73 (1.23)	13.78 (1.11)	0.193	0.04
Sedentary (min/day)	584.09 (79.34)	560.99 (79.13)	605.29 (73.46)	<0.001	0.58
Light-intensity PA (min/day)	181.72 (54.94)	190.09 (55.52)	174.04 (53.31)	<0.001	0.29
MVPA (min/day)	59.39 (31.55)	72.61 (34.42)	47.25 (22.71)	<0.001	0.87
**School hours (8:00 a.m.–2:45 p.m.)**					
Wear time (h/day)	6.20 (0.54)	6.22 (0.56)	6.18 (0.52)	0.656	0.06
Sedentary (min/day)	266.50 (39.93)	257.27 (40.19)	274.97 (37.79)	<0.001	0.45
Light-intensity PA (min/day)	77.78 (25.00)	81.84 (24.53)	74.05 (24.86)	<0.001	0.32
MVPA (min/day)	27.95 (16.35)	34.18 (18.53)	22.23 (11.39)	<0.001	0.78
**Non-school hours**					
Wear time (h/day)	7.54 (0.98)	7.50 (1.04)	7.58 (0.92)	0.078	0.08
Sedentary (min/day)	317.59 (55.27)	303.73 (56.33)	330.32 (51.12)	<0.001	0.49
Light-intensity PA (min/day)	103.94 (35.14)	108.24 (36.7)	99.99 (33.19)	<0.001	0.24
MVPA (min/day)	31.43 (19.35)	38.42 (21.74)	25.02 (14.10)	<0.001	0.73

PA = physical activity; MVPA = moderate and vigorous-intensity physical activity. ^a^ *p*-Value is for the gender difference estimated from a three-level multilevel linear model accounting for the clustered sampling design (i.e., children nested within schools and school nested within regions). The comparisons of sedentary, light-intensity PA, and MVPA were adjusted for the respective total wear time. ^b^ Cohen’s *d* was calculated using means and standard deviation.

**Table 3:** The prevalence of objectively measured meeting MVPA recommendations.

	Total (*n* = 1053)	Boys (*n* = 504)	Girls (*n* = 549)	*p*-Value ^a^
**All-day MVPA recommendation (*n*, %)**				
Yes (≥60 MVPA min/day)	436 (41.41%)	297 (58.93%)	139 (25.32%)	<0.001
No (<60 MVPA min/day)	617 (58.59%)	207 (41.07%)	410 (75.68%)	
**School hours MVPA recommendation (*n*, %)**				<0.001
Yes (≥30 MVPA min/school hours)	395 (37.51%)	262 (51.98%)	133 (24.23%)	
No (<30 MVPA min/school hours)	658 (62.49%)	242 (48.02%)	416 (75.77%)	

MVPA = moderate and vigorous intensity physical activity. ^a^ *p*-value is for the gender difference estimated from a three-level multilevel generalized linear model accounting for the clustered sampling design (i.e., children nested within schools and school nested within regions).

**Table 4:** Self-reported physical activity pursuits and sedentary behaviors.

	Total (*n* = 1053)	Boys (*n* = 504)	Girls (*n* = 549)	*p*-Value ^a^
**Watching television (*n*, %)**				0.023
Not at all	175 (16.92%)	78 (15.82%)	97 (17.93%)	
<1 h/day	441 (42.65%)	202 (40.97%)	239 (44.18%)	
1 h/day	204 (19.73%)	89 (18.05%)	115 (21.26%)	
2+ h/day	214 (20.70%)	124 (25.15%)	90 (16.64%)	
Missing	19 (-)	11 (-)	8 (-)	
**Computer uses (*n*, %) ^b^**				<0.001
Not at all	523 (50.43%)	225 (45.45%)	298 (54.98%)	
<1 h/day	246 (23.72%)	92 (18.59%)	154 (28.41%)	
1 h/day	133 (12.83%)	81 (16.36%)	52 (9.59%)	
2+ h/day	135 (13.02%)	97 (19.60%)	38 (7.01%)	
Missing	16 (-)	9 (-)	7 (-)	<0.001
**Sports team participation (*n*, %)**				
No sports team/yr	366 (35.33%)	124 (25.15%)	242 (44.57%)	
1 or more sports teams/yr	670 (64.67%)	369 (74.85%)	301 (55.43%)	
Missing	17 (-)	11 (-)	6 (-)	
**Self-reported meeting MVPA recommendation (*n*, %)**				<0.001
Yes (≥60 MVPA min/day)	337 (32.50%)	190 (38.46%)	147 (27.07%)	
No (<60 MVPA min/day)	700 (67.50%)	304 (61.54%)	396 (72.93%)	
Missing	16 (-)	10 (-)	6 (-)	

Values are *n* (%). MVPA = moderate and vigorous-intensity physical activity. ^a^ *p*-Value is for the gender difference estimated from a three-level multilevel linear model accounting for the clustered sampling design (i.e., children nested within schools and school nested within regions). ^b^ time spent using a computer including playing on a computer or video games on an average school day.

**Table 5:** A prediction of objectively measured meeting all-day MVPA recommendation ^a^.

	Total	Boys	Girls
Weight status (obese)	0.94 (0.69, 1.28)	0.92 (0.59, 1.43)	1.08 (0.70, 1.68)
Self-reported meeting ≥60-min MVPA/day	**1.37 (1.01, 1.86)**	1.45 (0.93, 2.27)	1.28 (0.82, 2.00)
Participating 1+ sport teams/yr	**1.40 (1.03, 2.90)**	**1.72 (1.08, 2.73)**	1.14 (0.75, 1.73)
Watching TV 2+ h/day	1.23 (0.88, 1.72)	1.33 (0.84, 2.12)	1.14 (0.67, 1.94)
Using computer 2+ h/day	0.96 (0.64, 1.45)	0.68 (0.41, 1.13)	2.01 (0.98, 4.10)

Values are odds ratios (95% confidence intervals). ^a^ A three-level multilevel generalized linear model was constructed predicting the likelihood of meeting objectively measured MVPA recommendation (≥60 min of MVPA per day). The gender was further adjusted in the model with total sample. Bold indicates statistically significant at alpha level of 0.05.

## Data Availability

Y.K. stored all data supporting the reported results. Requests for data should be directed to Y.K.
